# Effects of Paprika Xanthophyll Intake on Endurance and Cognitive Function in College Students: A Crossover Randomized Controlled Trial

**DOI:** 10.3390/nu17172780

**Published:** 2025-08-27

**Authors:** Donghyun Kim, Tsuyoshi Wadazumi

**Affiliations:** 1 Graduate School of Health and Well-Being, Kansai University, Osaka 590-8515, Japan; kdh3970@gmail.com; 2 Faculty of Health and Well-Being, Kansai University, Osaka 590-8515, Japan

**Keywords:** xanthophyll, deformability, oxygen delivery efficiency, heart rate, cognitive function

## Abstract

Background/Objective: Paprika xanthophylls (PXs) have potent antioxidant properties and are believed to improve oxygen delivery (DO_2_) efficiency by enhancing red blood cell (RBC) deformability. This study investigated whether PX ingestion improves endurance performance and subsequently enhances cognitive function by improving brain microcirculation. Methods: A crossover design was used to compare the effects of PX ingestion and a control condition in 21 healthy college students (18 males, 3 females). Each participant served as their own control, completing both conditions in a randomized order with a one-month washout period to eliminate any carryover effects. The participants underwent an incremental load test, a constant load test, the Trail Making Test Type B (TMT-B), and the Stroop test (ST). Results: In the incremental tests, the PX group showed a significantly lower heart rate (*p* = 0.032) and higher exercise efficiency (EE) (*p* = 0.004). In the constant load test, heart rate was lower (*p* = 0.020), and EE was higher (*p* = 0.030). No significant between-group differences were found in the cognitive tests; however, the PX group showed significant improvements in the TMT-B (*p* = 0.034) and ST interference rate I (*p* = 0.040). Conclusions: It is speculated that PX intake may improve DO_2_ efficiency, which could contribute to the observed enhancements in endurance performance and, in turn, positively affect cognitive function by optimizing the brain’s oxygenation state. However, due to the absence of a placebo control group and unmeasured RBC deformability and cerebral blood flow, as well as a significant male predominance, this study’s results should be interpreted with caution.

## 1. Introduction

Endurance exercise performance largely depends on the efficiency of oxygen delivery (DO_2_) and oxygen utilization. Oxygen is crucial for aerobic energy production, and its efficiency is evaluated by maximal oxygen uptake (VO_2_ max), anaerobic threshold (AT), and exercise efficiency (EE) [1,2]. Red blood cell (RBC) deformability and microcirculation are deeply involved in DO_2_ efficiency; if these are impaired, oxygen diffusion is inhibited, leading to a decrease in endurance capacity [3,4,5].

Recently, interventions with antioxidant nutrients, aimed at improving DO_2_ efficiency, have garnered attention. Several studies on humans have shown that paprika xanthophyll (PX) found in red paprika possesses antioxidant properties similar to other xanthophylls, such as lutein [6] and zeaxanthin [6,7]. Notably, capsanthin and capsorubin, which are the primary xanthophylls in red paprika [8], are considered to exhibit high scavenging activity against reactive oxygen species [9]. Furthermore, these pigments have been confirmed to be contained in RBCs [10], suggesting the possibility that PX may contribute to a reduction in oxidative stress and improvement of DO_2_ efficiency. Furthermore, PX intake has been shown to reduce heart rate [11] and oxygen consumption [11,12] during exercise and improve EE [11], suggesting its potential to enhance endurance performance through improved DO_2_ efficiency.

Meanwhile, improvements in DO_2_ efficiency may extend beyond the muscular system to include cerebral microcirculation. Specifically, higher-order cognitive function areas, such as the dorsolateral prefrontal cortex (DLPFC) [13], are susceptible to cognitive decline due to insufficient oxygen supply [14,15]. Conversely, improved oxygenation has also been reported to enhance cognitive function [16,17,18]. However, while previous PX research has primarily focused on muscular performance and physiological responses [11,12], its effects on cerebral microcirculation and cognitive function have not been sufficiently investigated. A preliminary study [19] was suggestive of an effect but was limited by a small sample size and lacked key physiological markers to elucidate the mechanism.

The novelty of this study lies in its integrated verification of PX intake’s improvement in DO_2_ efficiency as a common physiological mechanism for both muscle and brain, examining both exercise performance and cognitive function aspects. This approach presents an interface between exercise physiology indicators and cognitive neuroscience (traditionally treated separately) and provides insights applicable to individuals without exercise habits and older adults as a non-training intervention.

This study hypothesized that improved DO_2_ efficiency through PX intake would not only enhance oxygen supply to muscles and improve endurance performance but also extend to cerebral microcirculation, thereby affecting higher-order cognitive functions, such as attention and executive function. Therefore, this study aimed to conduct a crossover randomized controlled trial in healthy college students to verify the improvement in endurance performance brought about by PX intake and its accompanying effects on neurocognitive function, using both physiological and cognitive function indicators.

## 2. Materials and Methods

### 2.1. Participants

An a priori power analysis was conducted using G*Power software (version 3.1.9.4) to determine the required sample size. The analysis utilized an F-test for a repeated-measures ANOVA (within-factors), specifically targeting the interaction effect of exercise and PX.

We aimed to detect a medium effect size (f = 0.25) with a statistical power of 80% (1 − β = 0.80) and a significance level of α = 0.05. Based on the existing literature, a correlation among the four repeated measures was assumed to be 0.6. The analysis confirmed that a total of 19 participants would be required. Our final sample size of 21 participants therefore provided adequate power for the study. All the participants were healthy university students aged 20–21 who engaged in recreational sports but were not athletes. Our study included 21 healthy college students males (*n* = 18, mean age 20.7 ± 0.6 years) and females (*n* = 3, mean age 20.3 ± 0.5 years), who were recruited from 28 to 30 May 2024. To ensure a homogeneous sample, individuals with a history of cardiovascular or metabolic diseases were to be excluded; however, as a result of the screening, no participants were excluded based on this criterion. Furthermore, to minimize the influence of physical activity level, individuals who engaged in regular endurance training or high-intensity interval training were also excluded. Their physical characteristics are shown in Table 1.

### 2.2. Experimental Protocol

We used the PX supplement to build upon the preliminary findings of a previous pilot study [19] that explored its potential efficacy. This follow-up investigation was designed to more clearly elucidate the underlying physiological mechanisms of PX and was conducted with corresponding markers. In this experiment, there were two conditions. This study was designed as a crossover randomized controlled experiment, comprising two trials: Trial 1 (CON) and Trial 2 (PX). The PX trial involved PX supplementation, while the CON trial was a state of no PX intake or a washout state. The PX supplement used was a plant-based soft capsule (manufactured by Sansei Yakuhin Co., Ltd., Shizuoka, Japan) containing 9 mg of paprika xanthophylls per capsule (PapriX-oil HP; Glico Nutrition Co., Ltd., Osaka, Japan). Although the specific ratio of each component is not publicly disclosed, the product is standardized by its total xanthophyll content. The supplement contained seven types of xanthophylls: capsanthin, cucurbitaxanthin A, β-cryptoxanthin, zeaxanthin, capsanthin epoxide, capsorubin, and cryptocapsin. The participants were instructed to take one capsule with their breakfast daily, with no specific restrictions on food or drink. Adherence to the regimen was verbally confirmed on a weekly basis. Although HPLC analysis was not conducted for this study, the product’s quality and bioavailability are well-supported by previous research [10], which confirmed the absorption of paprika xanthophylls into human plasma and erythrocytes.

**Figure 1:**
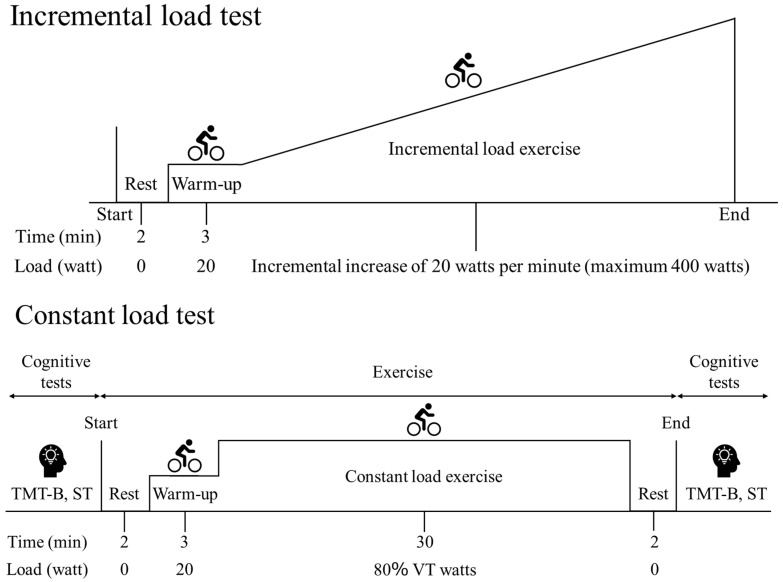
Experiment protocol. VT: ventilatory threshold; TMT-B: Trail Making Test Type B; ST: Stroop test.

The participants came to the laboratory at a pre-determined time to complete the experiment. They were instructed not to engage in strenuous exercise or drink alcohol the day before the experiment and not to consume anything other than water for four hours before the start of the measurement on the day of the measurement. The experiment was preceded by an incremental load test as a preliminary test to detect the ventilatory threshold (VT) of all the participants The incremental load test proceeded as follows (Figure 1): Begin the experiment and rest for 2 min, warm-up with a 20-watt load for 3 min, increase the load by 20 watts per minute, and maintain pedal speed at 60 rpm. The end point of the experiment was determined using the same method as in previous research [20].

**Figure 2:**
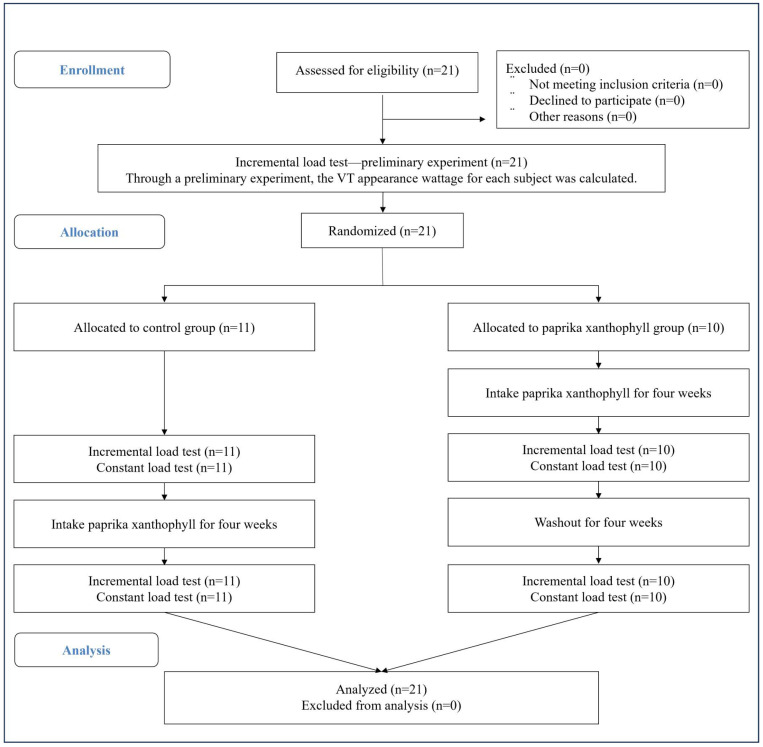
CONSORT flow diagram.

After the completion of the preliminary experiment, the participants were randomly assigned to the treatment order using Microsoft Excel’s RAND function, which generated a list of random numbers to determine the sequence. Given the non-placebo, crossover design, allocation concealment and blinding were not required for this study. Two types of exercise load tests were measured in both the CON and PX groups: an incremental load test and a constant load test. The CON group performed the incremental and constant load tests without PX intake, followed by at least one month of PX supplementation. One month of PX consumption was followed by the incremental and constant load tests as before PX consumption. Meanwhile, the PX group completed incremental and constant load tests after one month of PX intake. They subsequently underwent a one-month washout period, which, according to a previous study [10], was considered sufficient to minimize carry-over effects. After this, the incremental and constant load tests were conducted in the same manner (Figure 2). The incremental load test was performed using the same procedure as the preliminary test, with the VT watt detected from the preliminary test data, was used for the constant load test.

The procedure for the constant load test was as follows: Perform a pre-cognitive function test, followed by 2 min of rest and 3 min of warm-up at 20 W, followed by 30 min of constant load exercise at 80% of the VT calculated from the preliminary test data (Figure 1). The participants were instructed to maintain pedal speed at 60 rpm. The participants then rested for 2 min and completed the same cognitive function test (Figure 1). As cognitive function tests, the Trail Making Test Type B (TMT-B) was performed three times and a Stroop test composed of four conditions was administered. The design aimed to capture changes in cognitive function in the state of enhanced endurance exercise performance with PX and to assess the effects of DO_2_ efficiency under exercise load in an integrated manner. The exercise load was measured using an Aerobike 75XL III (KONAMI Sports Club Co., Ltd., Tokyo, Japan), expiratory gas data using an expiratory gas analyzer (Aeromonitor AE-310s MINATO medical science Co., Ltd., Osaka, Japan), and heart rate using an electrocardiograph (LX-7120 Fukuda Denshi Co., Ltd., Tokyo, Japan) and ECG monitor (DS-7520 Fukuda Denshi Co., Ltd., Tokyo, Japan). The measured data were recorded in AT for Windows (MINATO medical science Co., Ltd., Osaka, Japan).

### 2.3. Cognitive Task

Although various testing methods exist for evaluating cognitive function, we used the Japanese version of the New Stroop Test II (ST) and TMT-B, commonly used to measure executive function. The ST evaluates an individual’s ability to suppress interference from two pieces of information—letter meaning and letter color—and make an attentional choice. The interference includes Stroop interference (SI), which eliminates letter meaning and reads letter color, and reverse-Stroop interference (RI), which eliminates letter color and reads letter meaning.

The New ST-II [21] consists of four tasks: reverse Stroop control condition, reverse SI condition, Stroop control condition, and SI condition. The control condition matches letter color and meaning, and the interference condition mismatches letter color and meaning. Each task consists of 10 practice tasks and 100 main tasks. The main task is performed for one minute. The interference rate is calculated from the results, which confirm the Stroop effect that occurs in Tasks 2 and 4 (SI conditions: incongruent) compared with Tasks 1 and 3 (control conditions: congruent). We used the following formula for calculating the interference rate.

RI rate (Interference Rate I): [(number of correct answers for Task 1 − number of correct answers for Task 2)/number of correct answers for Task 1] × 100

SI rate (Interference Rate II): [(number of correct answers for Task 3 − number of correct answers for Task 4)/number of correct answers for Task 3] × 100

The TMT consists of two types of tests: TMT-A and TMT-B. TMT-A tests an individual’s ability to connect numbers from 1 to 25 in sequence and mainly requires a visual perception ability. The TMT-B primarily assesses working memory but also assesses cognitive flexibility [22]. We used the Japanese version of the TMT-B in this study.

The participants performed the cognitive function test on a Microsoft Surface Go 3 tablet by touching the screen. They practiced the TMT-B and ST several times beforehand.

### 2.4. Data Analysis and Statistical Processing

Data analysis for the incremental and constant load tests included watt, oxygen uptake (VO_2_), carbon dioxide output (VCO_2_), respiratory exchange ratio (RER), heart rate (HR), minute ventilation (VE), end-tidal pressure of carbon dioxide (PETCO_2_), and end-tidal pressure of oxygen (PETO_2_) recorded in AT for Windows. The VT detection time point was based on the criteria used in previous research [23]. We used the following detection methods as criteria for detection: (1) the point at which R begins to rise; (2) the point at which VCO_2_ rises relative to VO_2_; (3) the point at which VE/VCO_2_ does not rise and VE/VO_2_ increases; (4) the point at which VE rises relative to VO_2_; (5) the point at which PETCO_2_ does not change and PETO_2_ increases. Additionally, using data on watt, VO_2_, and VCO_2_ as indicators of EE and the same equation as in a previous study [24], we calculated the gross efficiency (GE).

The expiratory gas data from the incremental load test were analyzed in two ways. First, we divided the data into three ranges (R1: average warm-up value for each participant, R2: average value from the start of ramp load exercise to the time of VT appearance, and R3: average value from the time of VT appearance to the end of incremental load test) and then calculated and analyzed the average values. Second, the expiratory gas data at the time of VT emergence and peak were analyzed for each participant. The expiratory gas data from the constant load test were analyzed using 5 min average values. Pre- and post-TMT-B were measured three times for each of the pre- and post-tests. To reduce measurement error, the reliability for the average of the top two performances was calculated using ICC (1,2), and the reliability for the average of all three performances was calculated using ICC (1,3) [25]. The reliability for the average of the top two and of all three performances was 0.72 and 0.66, respectively. Both results are in the “substantial” range [26]. Since the average of the top two performances showed higher reliability, these scores were used as the data for analysis. ST was also analyzed by calculating interference rates I and II from the number of correct answers for each task. For statistical analysis, IBM SPSS Statistics 29 was used. The collected data were assessed for normality using the Shapiro–Wilk test and were presented as mean ± standard deviation (SD). Data from the incremental load test, constant load test, and cognitive function tests were analyzed using a two-way RM ANOVA for Group × Time interaction, with Bonferroni correction for post hoc comparisons. Additionally, expiratory gas data at the ventilatory threshold (VT) and peak exercise points for each participant were analyzed between pre- and post-intervention conditions within each group using paired *t*-tests. Effect sizes were analyzed using eta-squared (η^2^) values (where η^2^ ≥ 0.01 indicates a small effect, η^2^ ≥ 0.06 indicates a medium effect, and η^2^ ≥ 0.14 indicates a large effect) and Cohen’s d values (where d ≥ 0.2 indicates a small effect, d ≥ 0.5 indicates a medium effect, and d ≥ 0.8 indicates a large effect). All significance tests were conducted using two-tailed tests, with a *p*-value of less than 0.05 considered statistically significant.

## 3. Results

### 3.1. Incremental Load Test

**Figure 3:**
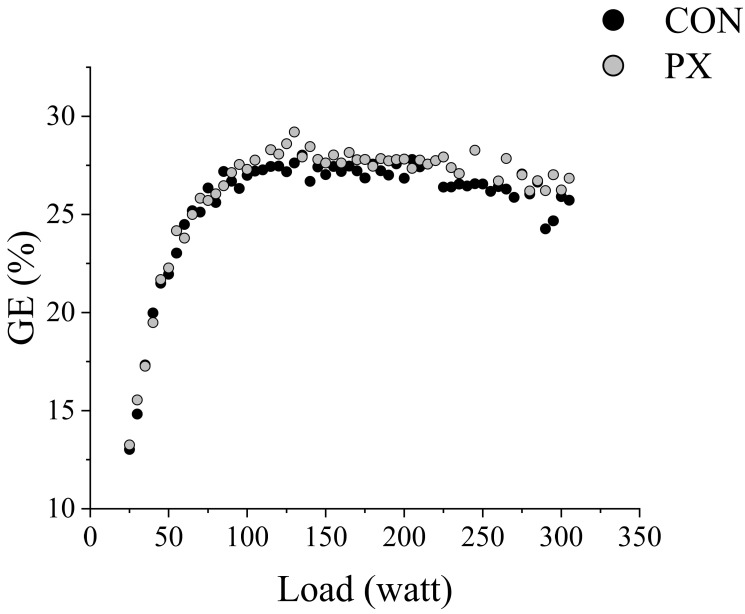
GE and watt during incremental load test and CON and PX trials. Abbreviations: GE: gross efficiency; %: percent; CON: control trial; PX: paprika xanthophyll trial; Load (watt): workload on a cycle ergometer.

**Figure 4:**
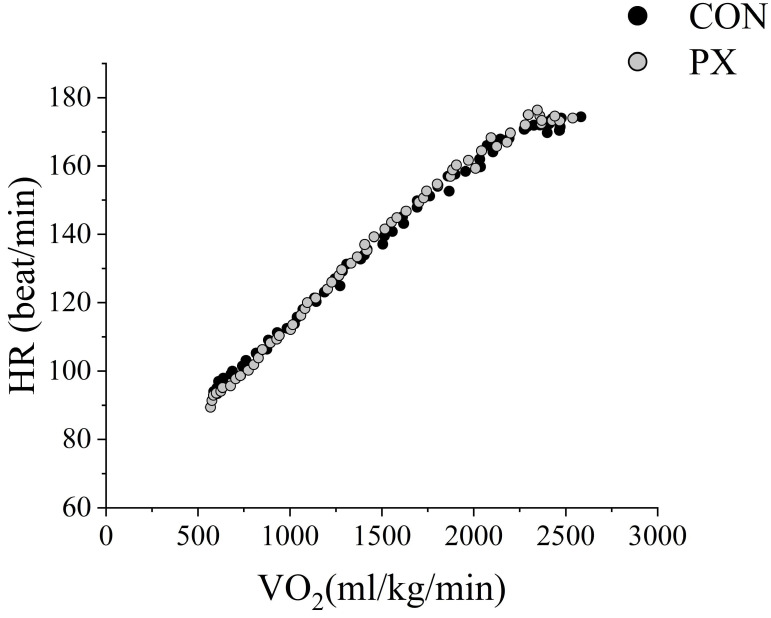
VO_2_ and HR during incremental load test and CON and PX trials. Abbreviations: HR: heart rate; min: minute; CON: control trial; PX: paprika xanthophyll trial; VO_2_: oxygen uptake; ml: milliliter; kg: kilogram.

For the incremental load test, the expiratory gas data were divided into three ranges. A two-way RM ANOVA revealed a significant Group × Time interaction for GE (F[1.49,29.87] = 7.65, *p* = 0.004, η^2^ = 0.277), indicating a large effect. Post hoc analysis indicated significant differences in GE between the CON and PX groups in Range 2 (*p* = 0.040) and Range 3 (*p* = 0.010) (Table 2) (Figure 3). In addition, no significant change was observed in the HR–VO_2_ relation before and after PX intake (Figure 4).

A significant main effect of Group was observed for GE (F[1,20] = 4.738, *p* = 0.042, η^2^ = 0.192), a large effect. Additionally, a significant main effect of Time was observed for VO_2_ (F[1.02,20.46] = 213.23, *p* = 0.000, η^2^ = 0.914), VCO_2_ (F[1.02,20.41] = 234.06, *p* = 0.000, η^2^ = 0.921), RER (F[1,20] = 286.68, *p* = 0.000, η^2^ = 0.935), HR (F[1.25,25.01] = 769.60, *p* = 0.000, η^2^ = 0.975), and GE (F[1.40,24.08] = 828.07, *p* = 0.000, η^2^ = 0.976) (Table 2), all of which showed extremely large effects.

A paired *t*-test conducted on each group’s data, based on the VT appearance watt of the PX group, showed that the PX group exhibited significantly lower HR values (t[20] = 2.298, *p* = 0.032, d = 0.502) compared to the CON group, a medium effect. For the peak data, paired *t*-tests revealed that GE (t[20] = −2.793, *p* = 0.011, d = −0.685) and watt (t[20] = −3.137, *p* = 0.005, d = −0.610) were significantly higher in the PX group compared to the CON group (Table 3), both representing medium to large effects.

### 3.2. Constant Load Test

For the constant load test, a two-way RM ANOVA revealed a significant Group × Time interaction was found for VO_2_ (F[2.89,57.88] = 3.30, *p* = 0.028, η^2^ = 0.142) and VCO_2_ (F[5,100] = 3.02, *p* = 0.014, η^2^ = 0.131), with both showing large effects. Post hoc analysis revealed significant differences between the CON and PX groups for several variables at various time points. For VO_2_, significant differences were found at 5 (*p* = 0.025), 10 (*p* = 0.025), and 15 min (*p* = 0.030). Significant differences were also observed for VCO_2_ at 5 (*p* = 0.043), 10 (*p* = 0.022), and 15 min (*p* = 0.037), and for GE at 5 (*p* = 0.005), 10 (*p* = 0.007), and 15 min (*p* = 0.011). Furthermore, significant differences were indicated for HR at 5 (*p* = 0.033), 10 (*p* = 0.020), 15 (*p* = 0.033), 20 (*p* = 0.016), 25 (*p* = 0.019), and 30 min (*p* = 0.030) (Table 4).

A significant main effect of Group was observed for HR (F[1,20] = 6.38, *p* = 0.020, η^2^ = 0.242) and GE (F[1,20] = 5.46, *p* = 0.030, η^2^ = 0.215), both large effects. Additionally, a significant main effect of Time was observed for VO_2_ (F[1.58,31.55] = 86.07, *p* = 0.000, η^2^ = 0.811), VCO_2_ (F[1.62,32.39] = 88.45, *p* = 0.000, η^2^ = 0.816), RER (F[1.80,36.09] = 42.63, *p* = 0.000, η^2^ = 0.681), HR (F[1.30,25.94] = 134.17, *p* = 0.000, η^2^ = 0.870), and GE (F[1.39,27.86] = 6.09, *p* = 0.012, η^2^ = 0.233), all of which showed extremely large effects.

### 3.3. Cognitive Function Test

For the cognitive function tests (TMT-B, ST), a two-way RM ANOVA was conducted to analyze the change in cognitive function in both groups for TMT-B, ST IR I, and ST IR II. The analysis revealed no significant Group × Time interaction for any of the variables (TMT-B: F[1,20] = 0.025, *p* = 0.877, η^2^ = 0.001; ST IR I: F[1,20] = 0.483, *p* = 0.495, η^2^ = 0.024; ST IR II: F[1,20] = 0.315, *p* = 0.581, η^2^ = 0.016), all of which showed small effects. Additionally, there was no significant main effect of Group (TMT-B: F[1,20] = 2.336, *p* = 0.142, η^2^ = 0.105, a medium effect; ST IR I: F[1,20] = 0.056, *p* = 0.816, η^2^ = 0.003, a small effect; ST IR II: F[1,20] = 1.120, *p* = 0.303, η^2^ = 0.053, a small effect).

Regarding the main effect of Time, a significant improvement was observed for ST IR I (F[1,20] = 6.615, *p* = 0.018, η^2^ = 0.249), which indicated a large effect. In contrast, the main effect of Time was not significant for TMT-B (F[1,20] = 3.879, *p* = 0.063, η^2^ = 0.162), which showed a large effect, or ST IR II (F[1,20] = 0.307, *p* = 0.585, η^2^ = 0.015), which showed a small effect. Post hoc analysis confirmed significant pre- to post-intervention improvements for both the TMT-B (*p* = 0.034) and ST interference rate I (*p* = 0.040) only within the PX group (Table 4).

## 4. Discussion

We hypothesized that PX intake improves DO_2_ efficiency, leading to enhanced endurance exercise performance and cognitive function by boosting microcirculatory function in both muscles and the brain. To investigate this, this study aimed to clarify how PX intake improves endurance performance and the subsequent impact of this state on cognitive function. Based on these findings, we speculate that PX intake not only improves DO_2_ efficiency in the muscular system but also enhances the “quality” of oxygen supply to the brain. This could explain the observed benefits for both endurance performance and cognitive function.

### 4.1. Mechanisms of Endurance Performance Enhancement by PX Intake

Improvements in endurance performance have traditionally been explained by enhanced cardiorespiratory function, increased VO_2_ max, and increased peripheral tissue oxygen utilization (ERO_2_: oxygen extraction ratio) attributable to training [2,27]. However, the PX group showed significant improvements in GE, watts at VT appearance, and watts at maximal load, with no significant change in VO_2_ peak (Figure 3). These results suggest that PX ingestion may have improved DO_2_ efficiency without an increase in oxygen intake, thus increasing the limits of aerobic metabolism.

In this study, it is speculated that PX intake improved DO_2_ through mechanisms such as suppressing oxygen consumption [12] and maintaining RBC deformability [9]. This led to significant improvements in both GE and the load at VT appearance (Table 3). We defined DO_2_ efficiency as “the efficient distribution and diffusion of oxygen in response to metabolic demand.” Moreover, we positioned this as a concept that indicates not only the size of DO_2_ but also the qualitative aspects of oxygen delivery, including the optimal oxygen delivery (or transport) manner at the active site—the efficiency of local blood flow regulation (distribution) and capillary-to-tissue transfer (diffusion). This definition is also consistent with previous studies focusing on DO_2_ quality [28,29], which is the main factor for the improvement in endurance performance in our study.

DO_2_ refers to the “delivered oxygen” per unit time [28], and VO_2_ peak is a composite index that, in addition to DO_2_, also reflects ERO_2_ in peripheral tissues [27]. This relation is expressed by the following equation: VO_2_ = DO_2_ × ERO_2_ (1). In other words, VO_2_ is determined by the product of how much oxygen can be carried (DO_2_) and how much oxygen can be used (ERO_2_). Therefore, even if the VO_2_ peak does not change, if the “way” of DO_2_—DO_2_ efficiency—is improved, a state in which energy supply by aerobic metabolism is maintained predominantly at the same VO_2_ even at higher exercise intensity can be established, and endurance performance can be supported. DO_2_ is defined by the product of HR, stroke volume (SV), and arterial blood oxygen content (CaO_2_) [1], expressed as DO_2_ = HR × SV × CaO_2_ (2). Furthermore, cardiac output (CO) is defined as the product of HR and SV (CO = HR × SV) (3), and with this relation, DO_2_ can also be expressed as CO × CaO_2_ (4).

In the present study, HR decreased significantly (Table 4), whereas VO_2_ did not change significantly (Figure 3). This indicates a situation in which HR decreased while DO_2_ was maintained, suggesting that CO may have been maintained without a decrease due to increased SV or improved DO_2_. These interpretations are indirect because SV was not directly measured in this study. However, PX ingestion may have promoted blood flow optimization and oxygen distribution efficiency at the capillary level and established DO_2_ with less HR dependence. In other words, a physiological state in which DO_2_ was maintained without increasing HR was realized, and DO_2_ to peripheral tissues was sufficiently secured.

In addition, we found no change in the HR–VO_2_ relation before and after PX ingestion (Figure 4). In general, when ERO_2_ in peripheral tissues is improved by endurance training, more VO_2_ is possible at the same HR. As such, the HR–VO_2_ relation is known to shift to the right [2,27,30]. However, we observed no such shift in the present study. The performance gains from PX ingestion (Figure 3) may have been achieved through improved DO_2_ efficiency rather than enhanced ERO_2_. This finding proposes a novel physiological mechanism for improving endurance, viewed from the perspective of optimizing oxygen transport independent of VO_2_ max and ERO_2_. It also represents significant insight into the potential of non-training approaches through nutritional supplement intervention.

This improvement in DO_2_ efficiency may involve microcirculation optimization, particularly the maintenance of erythrocyte deformability. RBCs maintain blood flow while flexibly deforming as they pass through capillaries 3–8 µm in diameter and are responsible for DO_2_ to peripheral tissues [4]. However, when oxidative stress peroxidizes lipids in erythrocyte membranes, deformability is reduced, causing increased blood flow resistance and inhibition of oxygen diffusion [5]. Capsanthin and capsorbin in PX have strong antioxidant properties and have been reported to contribute to maintaining deformability by inhibiting oxidation of the erythrocyte membrane [10]. This action may have supported DO_2_ to peripheral tissues via maintenance of blood flow at the capillary level and increased oxygen diffusion efficiency.

In addition, oxygen diffuses through the capillary wall into tissues, the efficiency of which is mainly defined by the oxygen partial pressure gradient and diffusion distance [28,31]. PX intake may maintain adequate capillary blood flow, resulting in an improved oxygen transport environment in muscle tissues and increased DO_2_ efficiency. Thus, optimization of oxygen delivery may have avoided local oxygen deprivation and enabled sustained aerobic metabolism.

Although RBC deformability and lactate concentration were not directly measured in this study, the decrease in HR during the constant load exercise (Table 4) and increase in watts during VT appearance (Table 3) can be interpreted as indirect evidence of improved DO_2_ efficiency. Ichihara et al. (2018) also reported a reduction in heart rate and oxygen consumption with PX ingestion [12], which is consistent with the results of our study.

Of particular note is the significant increase in VT emergence load in the PX group. VT is the transition point from aerobic to anaerobic metabolism and corresponds to the critical point at which lactate accumulation begins. With increased DO_2_ efficiency owing to PX intake, the same VO_2_ can be used to sustain higher-intensity exercise aerobically, and VT could be delayed. This is consistent with the findings of Faulhaber et al. (2021), who reported that improved DO_2_ maintains the aerobic metabolic advantage by promoting oxidative phosphorylation and suppressing lactate production [32]. Although lactate concentration was not measured in this study, a significant increase in exercise workload based on measured VT was observed, which can be evaluated as an indirect indicator suggesting an improvement in DO_2_ efficiency.

On the other hand, since there was no change in VO_2_ peak, it is improbable that PX directly increased mitochondrial oxygen utilization (oxidative phosphorylation capacity). Therefore, the improvement in endurance exercise performance in this study was most likely achieved by improving DO_2_ efficiency through the maintenance of peripheral circulation, particularly erythrocyte deformability and optimization of blood flow at the capillary level. In other words, PX ingestion did not increase the “total amount” (quantity) of oxygen but rather optimized the “way it is carried” (quality), which likely supported sustained aerobic metabolism and, consequently, endurance performance.

### 4.2. Effects on Cognitive Function and Quality of DO_2_

The improvement in DO_2_ efficiency (quality of DO_2_) due to PX intake may have extended not only to muscle tissue but also to brain function. The brain accounts for approximately 20% of the total metabolic rate of the entire body [33] and requires an uninterrupted supply of oxygen. In particular, the dorsolateral prefrontal cortex (DLPFC) region is involved in executive functions [13], and activation of the DLPFC has a high oxygen demand [34]. In this region, the “quality” of local DO_2_ in response to metabolic demand, i.e., maintenance of blood flow at the capillary level, oxygen partial pressure gradients, and oxygen diffusion efficiency, are more important for maintaining neural activity than the total amount of DO_2_ [29].

In this study, the PX group showed a significant increase in GE (Table 2 and Table 3) and ventilatory threshold (VT) onset load (Table 3) during the incremental load test and a decrease in heart rate during the constant load test (Table 4), suggesting an optimization of whole-body DO_2_ efficiency. A key feature of this study is that cognitive function tests were conducted while this improved DO_2_ efficiency was observed throughout the body.

Thus, in this study, neither the Group × Time interaction nor the main effect of Group was statistically significant; however, within-group comparisons in the PX group showed significant improvements in cognitive task performance for the TMT-B and ST interference rate I (Table 4). These tasks evaluate executive functions, particularly attentional shifting and inhibitory control, and their successful execution has been shown to be strongly associated with neural activity in the dorsolateral prefrontal cortex (DLPFC) [21,35]. However, no similar improvements were observed in the CON group.

This finding suggests that the improvement in DO_2_ efficiency from PX intake may have enabled oxygenation that matched the metabolic demand in higher-order cognitive function areas, including the DLPFC. To maintain and improve brain function, what is crucial is not only the quantity of oxygen supplied but also the “optimization of oxygen supply = quality of DO_2_” that precisely responds to metabolic demands. It is highly probable that the improved DO_2_ efficiency due to PX intake enabled a DO_2_ supply commensurate with the temporarily increased oxygen demand of the brain due to exercise stimuli, contributing to the improved performance in the TMT-B and ST interference rate I. However, this interpretation remains speculative, as cerebral oxygenation was not directly measured in this study. In addition, the antioxidant properties of capsanthin and capsorubin in PX [9,10] may have supplementarily contributed to neuronal function by mitigating oxidative stress, a neuroprotective mechanism known from other carotenoids [36].

Meanwhile, no significant changes were observed in the ST interference rate II. This task requires integrated activity in a wide range of brain regions other than the DLPFC, including the visual cortex and anterior cingulate cortex [21]. Takahashi et al. (2020) and Hu et al. (2021) reported that high-intensity acute exercise increases extensive cerebral blood flow and has a positive effect on performance on the Stroop task [37,38]. The moderate exercise load (80% of VT) used in our study may not have resulted in sufficient activation of these regions. In addition, the baseline value for interference rate II was relatively low, at an average of 7.8% [39]; as such, the possibility of a ceiling effect, as pointed out by Ishihara (2021), cannot be ruled out [40]. However, these interpretations are indirect because cerebral blood flow and oxygenation were not directly measured in this study. Future studies should more directly examine the relation between DLPFC oxygenation and cognitive function by assessing regional oxygenation dynamics using functional Near-Infrared Spectroscopy (fNIRS) or other methods. From these results, PX intake may have enabled enhanced whole-body DO_2_ efficiency and contributed to improved attention and executive function by achieving optimal oxygenation in the DLPFC, which responded to metabolic demands. This finding is new physiological evidence that links exercise physiology and cognitive neuroscience.

### 4.3. Novelty of This Study

While previous research hinted at PX’s potential to enhance endurance performance [11,12], the carry-over effects on cognitive function in an enhanced performance state, along with their underlying physiological mechanisms, remained largely unexplored.

This research indicates that PX intake may simultaneously improve both endurance performance and executive function by enhancing microcirculatory function in muscles and the brain, thereby increasing DO_2_ efficiency. Such a combined effect is significant as a novel ergogenic strategy that operates through a common mechanism of DO_2_ efficiency, differing from traditional training approaches that rely on increasing VO_2_ peak.

Furthermore, the achievement of improved endurance with PX without requiring exercise training suggests a non-invasive and practical nutritional intervention for sedentary individuals and older adults [18]. Additionally, this study’s unique attempt to indirectly evaluate the functional oxygenation of the DLPFC and cognitive function changes, based on improved DO_2_ efficiency, distinguishes it from existing research.

In summary, this study provides new insights into the fields of sports nutrition and neurofunction by demonstrating the potential of PX intake to simultaneously enhance endurance exercise performance (EE, VT) and cognitive function (TMT-B, ST) through improved microcirculatory function.

### 4.4. Limitations and Strengths

We did not directly measure erythrocyte deformability or cerebral blood flow, so causal relations regarding the effects of PX ingestion on microcirculation and DO_2_ efficiency, as well as on cognitive function, can only be interpreted indirectly. In particular, direct assessment of erythrocyte deformability and regional cerebral blood flow is required to clarify the dynamics of DO_2_ in muscle and brain tissues.

In this study, we did not include a placebo control group, which is a major methodological limitation and restricts the rigorous verification of PX-specific effects. The absence of a placebo makes it difficult to definitively rule out a potential placebo effect or other confounding factors that may have influenced our outcomes. Since the participants were aware they were receiving a supplement, a psychological “expectancy effect” may have influenced their performance, making it difficult to attribute the observed changes solely to the physiological effects of the supplementation. Additionally, it is difficult to definitively conclude that learning effects from repeated measurements were absent. However, a strength of our study is the adoption of a crossover design, where each participant served as their own control. This approach minimized the influence of individual differences and expectation bias.

Another major limitation of this study is the lack of an independent HPLC analysis of the PX supplement. While the manufacturer confirms the xanthophyll composition through internal quality control, detailed analytical data are not publicly available. Therefore, the complete elimination of uncertainty regarding the exact chemical composition of the product tested in this study is not possible. This point should be considered when interpreting the results of the present study.

Another limitation of this study is the small sample size (*n* = 21) and the significant male predominance (18 males vs. 3 females). This imbalance not only makes it difficult to analyze sex-specific effects but also suggests that the differences in BMI and potential variations in nutritional status between males and females limit the generalizability of our findings, which should therefore be interpreted with caution and may not be applicable to the female population.

### 4.5. Prospects

To address the limitations of the present study, long-term follow-up research is needed to establish the universal applicability and practical utility of PX intake. These studies should include direct physiological indicators, such as cerebral blood flow (e.g., fNIRS) and endothelial function (e.g., FMD), in diverse populations, including young adults, middle-aged and older individuals, and both physically active and sedentary individuals.

## 5. Conclusions

It is speculated, based on these findings, that PX intake may improve DO_2_ efficiency, possibly through the maintenance of RBC deformability and a reduction in blood flow resistance. This could be a new nutritional strategy to concurrently boost both endurance performance and cognitive function. Future research is needed to more clearly elucidate these mechanisms by directly assessing RBC deformability and localized cerebral blood flow.

## Figures and Tables

**Table 1:** Participants’ physical characteristics.

	Age (Years)	Height (cm)	Weight (kg)	BMI
Male (*n* = 18)	20.7 ± 0.6	172.1 ± 7.4	66.5 ± 10.0	22.3 ± 2.9
Female (*n* = 3)	20.3 ± 0.5	163.0 ± 6.0	47.9 ± 1.0	18.2 ± 1.3
Total (*n* = 21)	20.7 ± 0.6	169.0 ± 7.2	62.7 ± 9.0	21.9 ± 2.7

**Table 2:** Expiratory gas data during incremental exercise test (range).

Incremental Load Test (Range)															
		CON	PX	Main Effect: Group				Main Effect: Time				Interaction: Group × Time			
		Mean ± SD	Mean ± SD	F	df	*p*	η^2^	F	df	*p*	η^2^	F	df	*p*	η^2^
VO_2_ (mL/kg/min)	R1	525.8 ± 66.6	518.8 ± 57.0	0.860	1, 20	0.365	0.041	213.226	1.023, 20.460	0.000 ***	0.914	0.150	1.212, 24.249	0.750	0.007
	R2	925.5 ± 191.6	909.9 ± 161.0												
	R3	1911.5 ± 494.2	1894.6 ± 476.0												
VCO_2_ (mL/kg/min)	R1	443.6 ± 60.3	442.0 ± 50.9	0.367	1, 20	0.551	0.018	234.058	1.021, 20.413	0.000 ***	0.921	0.749	1.238, 24.766	0.423	0.036
	R2	834.2 ± 173.5	835.1 ± 156.2												
	R3	2195.7 ± 579.6	2223.5 ± 568.3												
RER	R1	0.85 ± 0.06	0.85 ± 0.06	1.539	1, 20	0.229	0.071	286.683	1, 20	0.000 ***	0.935	0.830	1, 20	0.443	0.040
	R2	0.89 ± 0.06	0.91 ± 0.06												
	R3	1.14 ± 0.09	1.16 ± 0.08												
HR (beat/min)	R1	88.5 ± 11.5	86.2 ± 9.3	1.408	1, 20	0.249	0.066	769.597	1.250, 25.010	0.000 ***	0.975	1.633	1.468, 29.358	0.215	0.075
	R2	108.7 ± 10.3	107.3 ± 10.3												
	R3	159.0 ± 11.6	159.0 ± 11.9												
GE (%)	R1	11.8 ± 1.6	11.7 ± 1.6	4.738	1, 20	0.042 *	0.192	828.067	1.404, 24.080	0.000 ***	0.976	7.645	1.493, 29.866	0.004 **	0.277
	R2	**24.0 ± 1.7 ^a^**	24.5 ± 1.7												
	R3	**26.9 ± 1.7 ^a^**	27.7 ± 1.4												

Note. * *p* < 0.05, ** *p* < 0.01, *** *p* < 0.001; post hoc test (Bonferroni), ^a^ *p* < 0.05, vs. PX. Abbreviations: CON: control trial; PX: paprika xanthophyll trial; SD: standard deviation; R1: average during warm-up; R2: average from exercise start to ventilatory threshold; R3: average from ventilatory threshold to exercise end; VO_2_: oxygen uptake; VCO_2_: carbon dioxide output; RER: respiratory exchange ratio; HR: heart rate; GE: gross efficiency; F: F-statistic; df: degrees of freedom; *p*: *p*-value; η^2^ (eta-squared): a measure of ANOVA effect size.

**Table 3:** Expiratory gas data during incremental exercise test (point).

Incremental Load Test (Point)								
	VT Watt							
	CON		PX		Paired *t*-Test			
	Mean	SD (±)	Mean	SD (±)	t	df	*p*	Cohen’s d
VO_2_ (mL/kg/min)	1314.6	334.4	1377.3	316.0	−2.179	20	0.041 *	−0.475
VCO_2_ (mL/kg/min)	1258.8	321.1	1382	355.5	−3.493	20	0.002 **	−0.762
RER	0.96	0.09	1.00	0.08	−1.561	20	0.134	−0.341
HR (beat/min)	127.9	11.8	130.5	12.3	−1.775	20	0.091	−0.387
GE (%)	26.8	2.2	27.5	2.0	−1.508	20	0.147	−0.329
Load (watt)	124.1	34.8	134.6	34.7	−5.911	20	0.000 ***	−1.290
	VT watt (Data with the same watt based on PX’s VT)							
	CON		PX		Paired *t*-test			
	Mean	SD (±)	Mean	SD (±)	t	df	*p*	cohen’s d
VO_2_ (mL/kg/min)	1388.2	377.0	1377.3	316.0	0.321	20	0.751	0.07
VCO_2_ (mL/kg/min)	1377.2	377.6	1382.0	355.5	−0.132	20	0.896	−0.029
RER	0.99	0.08	1.00	0.08	−0.133	20	0.896	−0.029
HR (beat/min)	133.6	13.0	130.5	12.3	2.298	20	0.032 *	0.502
GE (%)	27.6	2.2	27.5	2.0	0.181	20	0.858	0.04
Load (watt)	134.5	34.7	134.6	34.7	NA			
	Peak							
	CON		PX		Paired *t*-test			
	Mean	SD (±)	Mean	SD (±)	t	df	*p*	cohen’s d
VO_2_ (mL/kg/min)	2404.4	633.4	2345.7	637	1.011	20	0.324	0.221
VCO_2_ (mL/kg/min)	3101.9	823.9	3048.2	850.4	0.860	20	0.400	0.188
RER	1.29	0.12	1.29	0.10	−0.177	20	0.861	−0.039
HR (beat/min)	177.1	13.5	178.5	12.9	−1.191	20	0.248	−0.260
GE (%)	25.9	2.2	27.6	1.90	−2.793	20	0.011 *	−0.685
Load (watt)	233.5	57.2	243.1	60.9	−3.137	20	0.005 **	−0.610

Note: CON vs. PX; * *p* < 0.05, ** *p* < 0.01, *** *p* < 0.001. Abbreviations: VT: ventilatory threshold; CON: control trial; PX: paprika xanthophyll trial; SD: standard deviation; VO_2_: oxygen uptake; VCO_2_: carbon dioxide output; RER: respiratory exchange ratio; HR: heart rate; GE: gross efficiency; F: F-statistic; df: degrees of freedom; *p*: *p*-value; Cohen’s d: a measure of *t*-test effect size.

**Table 4:** Expiratory gas data during constant load test and cognitive task results.

Constant Load Test															
	Time (min)	CON	PX	Main Effect: Group				Main Effect: Time				Interaction: Group × Time			
		Mean ± SD	Mean ± SD	F	df	*p*	η^2^	F	df	*p*	η^2^	F	df	*p*	η^2^
VO_2_ (mL/kg/min)	5	**1138.9 ± 275.0 ^a^**	1104.5 ± 268.6	2.905	1, 20	0.104	0.127	86.073	1.58, 31.55	0.001 ***	0.811	3.304	2.89, 57.88	0.028 *	0.142
	10	**1243.5 ± 313.6 ^a^**	1201.9 ± 300.7												
	15	**1246.4 ± 305.1 ^a^**	1208.9 ± 301.4												
	20	1248.2 ± 306.4	1222.4 ± 306.2												
	25	1255.1 ± 310.4	1233.9 ± 312.7												
	30	1260.5 ± 310.3	1245.1 ± 308.8												
VCO_2_ (mL/kg/min)	5	**1089.8 ± 270.1 ^a^**	1049.6 ± 258.8	3.202	1, 20	0.089	0.138	88.452	1.62, 32.39	0.001 ***	0.816	3.020	5, 100	0.014 *	0.131
	10	**1246.7 ± 321.7 ^a^**	1197.2 ± 305.7												
	15	**1228.4 ± 312.6 ^a^**	1187.2 ± 302.6												
	20	1217.6 ± 312.6	1185.6 ± 307.4												
	25	1217.3 ± 315.1	1193.9 ± 315.3												
	30	1206.9 ± 309.5	1194.7 ± 314.2												
RER	5	0.95 ± 0.05	0.94 ± 0.04	0.059	1, 20	0.810	0.003	42.627	1.80, 36.09	0.001 ***	0.681	0.549	3.14, 62.77	0.658	0.027
	10	1.00 ± 0.04	1.00 ± 0.04												
	15	0.98 ± 0.04	0.98 ± 0.03												
	20	0.97 ± 0.04	0.97 ± 0.03												
	25	0.97 ± 0.04	0.97 ± 0.04												
	30	0.95 ± 0.04	0.96 ± 0.04												
HR (beat/min)	5	**119.1 ± 10.8 ^a^**	115.7 ± 9.3	6.382	1, 20	0.020 *	.242	134.169	1.30, 25.94	0.001 ***	0.870	1.586	2.34, 46.83	0.213	0.073
	10	**128.4 ± 13.2 ^a^**	123.2 ± 10.4												
	15	**131.7 ± 14.3 ^a^**	126.8 ± 11.2												
	20	**133.9 ± 15.1 ^a^**	128.3 ± 11.8												
	25	**135.6 ± 15.7 ^a^**	130.3 ± 12.4												
	30	**137.1 ± 15.9 ^a^**	132.0 ± 12.7												
GE (%)	5	**22.7 ± 1.9 ^a^**	23.4 ± 1.5	5.463	1, 20	0.030 *	0.215	6.086	1.39, 27.86	0.012 *	0.233	2.286	2.81, 56.24	0.092	0.103
	10	**21.8 ± 1.6 ^a^**	22.5 ± 1.4												
	15	**21.8 ± 1.8 ^a^**	22.5 ± 1.5												
	20	21.8 ± 1.8	22.3 ± 1.6												
	25	21.7 ± 1.8	22.2 ± 1.4												
	30	21.8 ± 2.0	22.1 ± 2.0												
Cognitive test															
	Time	CON	PX	Main Effect: Group				Main Effect: Time				Interaction: Group x Time			
		Mean ± SD	Mean ± SD	F	df	*p*	η^2^	F	df	*p*	η^2^	F	df	*p*	η^2^
TMT-B (s)	Pre	31.1 ± 7.7	**29.7 ± 4.7 ^b^**	2.336	1, 20	0.142	0.105	3.879	1, 20	0.063	0.162	0.025	1, 20	0.877	0.001
	Post	30.1 ± 5.3	28.6 ± 3.7												
ST IR I	Pre	12.6 ± 7.9	**13 ± 7.1 ^b^**	0.056	1, 20	0.816	0.003	6.615	1, 20	0.018 *	0.249	0.483	1, 20	0.495	0.024
	Post	9.6 ± 4.4	8.7 ± 6.8												
ST IR II	Pre	7.8 ± 6.4	7.7 ± 8.8	1.120	1, 20	0.303	0.053	0.307	1, 20	0.585	0.015	0.315	1, 20	0.581	0.016
	Post	6.0 ± 6.7	8.3 ± 6.3												

Note. * *p* < 0.05, *** *p* < 0.001; post hoc test (Bonferroni), ^a^ *p* < 0.05, vs. PX, ^b^ *p* < 0.05, vs. post. Abbreviations: min: minute; CON: control trial; PX: paprika xanthophyll trial; SD: standard deviation; VO_2_: oxygen uptake; VCO_2_: carbon dioxide output; RER: respiratory exchange ratio; HR: heart rate; GE: gross efficiency; TMT-B: Trail Making Test Type B; ST IR I, Stroop test interference rate I; ST IR II, Stroop test interference rate II; F: F-statistic; df: degrees of freedom; *p*: *p*-value; η^2^ (eta-squared): a measure of ANOVA effect size.

## Data Availability

Data generated or analyzed during this study are provided in full within the published article.

## Abbreviations

The following abbreviations are used in this manuscript:

DO_2_	Oxygen delivery
VO_2_ max	Maximal oxygen uptake
AT	Anaerobic threshold
EE	Exercise efficiency
RBC	Red blood cell
PX	Paprika xanthophyll
VT	Ventilatory threshold
CON	Control
TMT-B	Trail Making Test Type B
ST	New Stroop test Ⅱ
ST IR Ⅰ	Stroop test interference rate I
ST IR Ⅱ	Stroop test interference rate Ⅱ
VO_2_	Oxygen uptake
VCO_2_	Carbon dioxide output
RER	Respiratory exchange ratio
HR	Heart rate
VE	Minute ventilation
PETCO_2_	End-tidal pressure of carbon dioxide
PETO_2_	End-tidal pressure of oxygen
GE	Gross efficiency
SD	Standard deviation
ERO_2_	Oxygen extraction ratio
VO_2_ peak	Peak oxygen uptake
SV	Stroke volume
CaO_2_	Arterial blood oxygen content
CO	Cardiac output
DLPFC	Dorsolateral prefrontal cortex
